# Food safety challenges and One Health within Europe

**DOI:** 10.1186/s13028-017-0355-3

**Published:** 2018-01-03

**Authors:** Sofia Boqvist, Karin Söderqvist, Ivar Vågsholm

**Affiliations:** Department of Biomedical Sciences and Veterinary Public Health, Swedish University of Agricultural Sciences, PO Box 7036, 750 07 Uppsala, Sweden

**Keywords:** Antimicrobial resistance, Bovine spongiform encephalitis, Foodborne disease, Foodborne outbreak, *Listeria monocytogenes*, Norovirus, Raspberries, Shiga toxin-producing *Escherichia coli*, Sprouted seeds

## Abstract

This review discusses food safety aspects of importance from a One Health perspective, focusing on Europe. Using examples of food pathogen/food commodity combinations, spread of antimicrobial resistance in the food web and the risk of transmission of zoonotic pathogens in a circular system, it demonstrates how different perspectives are interconnected. The chosen examples all show the complexity of the food system and the necessity of using a One Health approach. Food safety resources should be allocated where they contribute most One Health benefits. Data on occurrence and disease burden and knowledge of source attribution are crucial in assessing costs and benefits of control measures. Future achievements in food safety, public health and welfare will largely be based on how well politicians, researchers, industry, national agencies and other stakeholders manage to collaborate using the One Health approach. It can be concluded that closer cooperation between different disciplines is necessary to avoid silo thinking when addressing important food safety challenges. The importance of this is often mentioned, but more proof of concept is needed by the research community.

## Background

Achievements in food safety, public health and welfare in coming decades will be based on successful One Health (formerly One Medicine) endeavours. One Health is a holistic or big-picture approach where the tenet is that welfare and wellbeing is based on human, animal and environmental health and that integration and sharing of information on animal and human health is the key to efficient health systems [1, 2]. One Health represents a rapidly growing range of synergistic disciplines, including food safety, public health, health economics, ecosystem health, social science and animal health, for addressing complex health problems [3]. Several zoonoses of public health importance in Europe are foodborne [4], but foodborne pathogens may also be non-zoonotic. What they have in common is that a One Health approach is needed to manage food safety and to understand the drivers and determinants for the emergence and persistence of human, animal and environmental threats.

Food safety resources should be allocated where they contribute most to One Health benefits. Without knowledge of, e.g. the incidence and burden of disease associated with particular pathogen/food commodity combinations, prioritisation of foodborne hazards against which mitigation measures should be put into force is difficult. Data on occurrence and disease burden are therefore crucial in assessing both benefits and costs of control measures. Moreover, there are challenges in prioritising among different public health risks when setting healthcare goals and supporting food safety and public health risk management by measuring burden of disease and source attribution [5–7].

Technical knowledge about pathogen transmission paths is important when designing control strategies against foodborne diseases but, for these methods to be efficient, consumer behaviour, food trends, economic incentives, trade and politics need to be taken into account [8]. Thus successful One Health policies build upon understanding the socio-economic contexts of farmers, food business operators and consumers. Moreover, a One Health approach is needed in efforts to reduce the amount of food waste and increase utilisation of nutrients, e.g. by using closed circular food systems [9].

This review examines food safety aspects of importance from a One Health perspective, focusing on Europe. A few examples are used to illustrate this and to depict the complexity of food webs. The examples also provide important lessons on future challenges and demonstrate the value and helpfulness of a One Health approach covering the entire food web.

## Search strategy

This literature review was provided through searches of PubMed (http://www.ncbi.nlm.nih.gov/pubmed), Google Scholar (https://scholar.google.com), Scopus^®^ (https://www.scopus.com) and Web of Science (https://apps.webofknowledge.com) using the key words and other terms relevant for this review (e.g. source attribution, DALY, QALY, cost-of-illness, community incidence), followed by evaluation of the bibliographies of relevant articles. Selection of the included papers were done stepwise. Initially the article titles from the literature searchers were assessed and if they were expected to be relevant for the paper the abstracts were read in the next step. If the abstracts were deemed relevant the full paper were retrieved and read. Web sites of relevant organizations and authorities (e.g. WHO, EFSA) were also used to retrieve information. Literature search was carried out between June and October 2017.

## Occurrence of zoonotic diseases and foodborne outbreaks within the EU

All member states within the European Union (EU) are obliged to collect data on occurrence of zoonoses, zoonotic agents, antimicrobial resistance, animal populations and foodborne outbreaks, according to Directive 2003/99/EC. These reports enable evaluation of trends and sources of zoonotic agents, antimicrobial resistance and foodborne outbreaks within the EU [4]. However, the data must be interpreted with caution because surveillance, monitoring and reporting are not harmonised within the EU, which contributes to substantial, but variable, underreporting.

*Campylobacter* spp. continues to be the most commonly reported zoonotic disease within the EU, followed by *Salmonella* [4, 10]. Similarly, according to the World Health Organisation (WHO) diarrhoeal disease agents contributed 49–68% of the total burden of foodborne disease in 2015, with non-typhoidal *S. enterica* and *Campylobacter* spp. being the most important bacterial pathogens [11]. It should also be noted that the human cases of zoonoses reported by the European Food Safety Authority (EFSA) only include zoonotic infections, and that data on the occurrence, sources and trends of other foodborne diseases are not included, such as diseases caused by *Cryptosporidium hominis*, norovirus and *Clostridium perfringens*. For example, Adak et al. [12] list *C. perfringens* as one of the most important foodborne pathogens, together with *Campylobacter* spp., *Salmonella*, Shiga toxin-producing *Escherichia coli* (STEC) O157 and *Listeria monocytogenes*. In 2010–11, around 27,000 cases were estimated to be part of a waterborne outbreak caused by the parasite *C. hominis* in Sweden [13], which shows the public health importance of this pathogen.

Information based on foodborne outbreaks reported to the EFSA should be interpreted with caution, as outbreak investigation systems are not harmonised within the EU [4]. However, it appears that bacterial agents, particularly *Salmonella* and *Campylobacter* spp., caused most of the reported outbreaks with a known source in 2014 and 2015 [4, 10]. In addition, bacterial toxins (other than those produced by *Clostridium botulinum*) and caliciviruses, including norovirus, were common causes of reported outbreaks in those years. Norovirus caused the highest number of cases related to foodborne outbreaks amongst the total number reported [4].

## Community incidence of foodborne gastrointestinal infections within the EU

Reliable data on foodborne community disease incidences are important in assessing the impact of infections on health, setting priorities for development of control strategies and monitoring progress [12, 14]. However, reported data are mainly based on passive surveillance, which underestimates the true incidence. This, combined with underreporting and under-diagnosis, further impairs the reliability of reported incidences. Moreover, the rates of underreporting and under-diagnosis vary between infectious agents and between countries, for reasons such as differences in surveillance routines, pathogens investigated, differences in healthcare systems and healthcare use, and laboratory practices [15].

A few cohort studies have investigated the extent of underreporting and under-diagnosis and have produced multipliers for estimating the true number of cases for every case reported in national surveillance of diseases caused by foodborne gastrointestinal pathogens. This enables better comparisons between nationally reported incidences. For example, the multiplier for campylobacteriosis and salmonellosis in the EU, based on data from Swedish travellers, was 47 and 58, respectively. The highest multiplier for campylobacteriosis was reported for Bulgaria (40,000 for each reported case) and for salmonellosis for Portugal (2080 for each reported case) [16]. However, there were large uncertainties in the dataset analysed in previous cohort studies. Moreover, changes to healthcare and reporting systems may change the multiplier. Studies in the United Kingdom showed that the multiplier increased from 3.2 to 4.7 for *Salmonella* and from 7.6 to 9.3 for *Campylobacter* spp. between 1999 and 2012 [17, 18]. Possible explanations include reduced general practitioner (GP) consultations due to changes in primary care and introduction of telephone advice services [18].

As cohort studies are time-consuming and complicated to perform, other studies have calculated community incidence of gastroenteritis by reconstructing surveillance pyramids to estimate different measures of disease at different levels in the surveillance system [14, 15]. It has also been reported that the country-specific multipliers for different EU countries vary between 9.3 and 100 for *Campylobacter* spp. and between 6.7 and 50 for *Salmonella* [15]. Thus, the reported number of cases, or reported incidence, only constitutes a fraction of the true incidence.

Disease surveillance priorities also vary between different countries within the EU. For example, toxoplasmosis is not considered a top priority in many countries due to the low number of reported cases. However, in the Netherlands, *Toxoplasma gondii* is mentioned as one of two pathogens (the other being *Campylobacter* spp.) for which specific prevalence targets in foodstuffs should be implemented [16].

## Contribution of various food sources to foodborne disease

Source attribution or pathogen account is an important tool for quantifying the contribution of various food sources to foodborne disease, thereby supporting food safety and public health management and intervention strategies [5, 19]. For food safety policy, it is important also to know the fraction that is attributable to sources other than food, for example environmental exposure, direct animal contact and human-to-human contact [20].

Control strategies for the major reservoirs will prevent subsequent human exposure, regardless of the transmission route or vehicle. However, the reservoirs or major sources of human foodborne illness may change over time. For example, at the end of the 1980s, broiler meat was the attributed major source of human salmonellosis in Denmark, but during the 1990s this changed to pork and later to table eggs [21]. In 1999, 47% of salmonellosis cases in Denmark were attributed to table eggs [5]. Since then, there have been EU baseline studies on *Salmonella* in laying hens, followed by implementation of EU-wide control programmes for *Salmonella* in laying hen systems (Commission Regulation (EU) No. 517/2011), resulting in a substantial reduction in *Salmonella* in laying hens [4]. This control programme has resulted in an approximately 50% reduction in the risk of Swedish travellers in the EU contracting salmonellosis [22] and there has also been a substantial reduction in reported human cases of salmonellosis [4]. There has been convergence amongst all EU Member States to the level of control achieved by Sweden and Finland, which implemented control programmes against *Salmonella* in food-producing animals as early as the 1960s [23, 24].

In the Netherlands, about two-thirds of the foodborne disease burden has been reported to be attributable to foodborne infections of animal origin, followed by human-to-human transmission and environmental transmission [16]. Similarly, Adak et al. [12] found that between 63 and 99% of STEC O157, *Campylobacter* spp., *L. monocytogenes* and non-typhoidal *Salmonella* cases were foodborne. The relatively high burden of environmental transmission was mainly attributable to *T. gondii*.

## Impact measurements of foodborne disease

Public health burdens of disease can be measured in several complementary ways, for example by using disability adjusted life years (DALY), quality adjusted life years (QALY) and cost-of-illness. Both DALY and cost-of-illness enable more comprehensive comparisons of infectious pathogens with different patterns of incidence and outcome, and show relatively little difference in ranking of pathogens [6, 7]. This can help policymakers allocate appropriate resources for food safety control and intervention efforts.

A recent report from the WHO investigating the burden of foodborne diseases showed that the DALY per 100,000 population for three European regions, together covering the whole of Europe, ranged from 24 to 28 for diarrhoeal agents to 10–19 for invasive infectious disease agents, 0.4–6 for helminths and 0.9–2 for chemicals and toxins [11].

In a study on disease burden in the Netherlands, the highest incidences were estimated for norovirus, rotavirus and bacterial toxins (*Staphylococcus aureus*, *C. perfringens*) [16]. However, on a yearly level the disease burden using DALY was highest for congenital *T. gondii* (23 DALY/100,000 population), followed by *Campylobacter* spp. (20 DALY/100,000 population), Rotavirus (11 DALY/100,000 population) and norovirus (9 DALY/100,000 population) [6, 16]. Norovirus and *Campylobacter* spp. were associated with the highest cost on a population level [6].

Cost-of-illness from a societal perspective includes the costs related to the healthcare sector (direct costs), resources used by patients and their families and non-healthcare-related resources (indirect costs) used, e.g. productivity losses due to absence from work, permanent or long-term disability or premature mortality [6]. The indirect costs are often much higher than the direct healthcare costs [7]. This was shown, e.g. in a *Salmonella* outbreak in the Netherlands in 2012, where the productivity losses were the main cost driver [25]. Variations in cost inventory methods make it difficult to interpret and compare costs across multiple studies and it has been suggested that a more standardised cost inventory would simplify the analysis [26].

From an economic perspective, it can be considered whether overall resources are used more efficiently by integrated, One Health surveillance than by a surveillance system with disconnected, sector-specific components. The lack of evidence detailing the costs and benefits to the different sectors of such collaborative efforts is one of the main hurdles to the wider adoption of One Health holistic approaches [27]. In one rare study, Martins et al. [28] reported increased costs using an integrated One Health system, although there were other intangible benefits.

On a population level, the highest costs are often reported for the most common foodborne infection, e.g. for campylobacteriosis [29]. However, the cost per case is often higher for diseases with relatively low burden and total societal costs, e.g. infection with *Campylobacter* spp. and STEC O157 due to sequelae such as reactive arthritis (RA), irritable bowel syndrome (IBS), Guillain–Barré syndrome (GB) and haemolytic uraemic syndrome (HUS) [29, 30].

Health-related benefits are generally more difficult to value than costs, so policymakers to date have largely relied on cost-effectiveness analysis to guide health policies [31]. Health effects are often quantified using QALY and interventions are evaluated by the cost per expected QALY gained, which is sometimes referred to as cost utility analysis [32]. By using DALY the disease burden to the society can be measured, and policymakers can then set the right priorities, while QALY is helpful for assessing benefits of interventions, selecting those that give most QALY for the money available [33].

The costs of controlling foodborne diseases in food-producing animals are high and some studies have investigated the effect that a relaxation of the Swedish *Salmonella* control programme in food-producing animals would have on public health and societal costs [34, 35]. They concluded that the number of reported domestic salmonellosis cases would increase substantially and the net cost effects would therefore be negative.

## Cases illustrating One Health and food safety in Europe

**Fig. 1:**
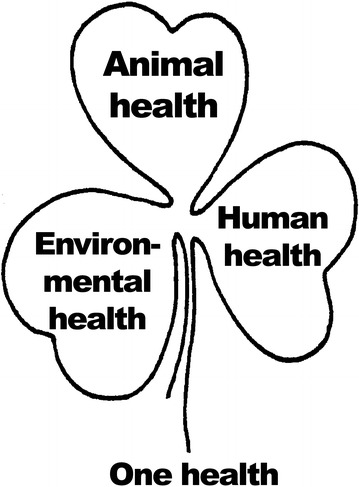
The key elements included in One Health

**Fig. 2:**
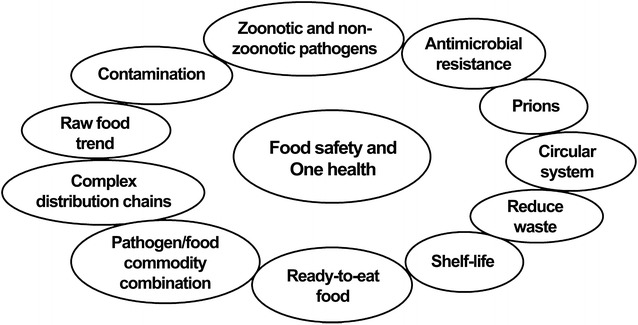
Key aspects related to the included cases illustrating food safety and One Health

The cases presented below provide more detailed insights into relevant sources and drivers from a One Health perspective (Fig. 1) and how these interact. Key aspects that are discussed in relation to the presented cases are illustrated in Fig. 2. To improve future food safety systems and increase the ability to respond to new and unknown food safety threats, we need to learn from the history. The chosen examples all show the complexity of the food system and the necessity of using a One Health approach.

## Pathogen/food commodity combinations and One Health challenges

### Norovirus-contaminated raspberries

The number of notifications for norovirus within the EU increased from 1998 to 2013 [36] and norovirus is currently the pathogen causing the highest number of cases of foodborne outbreaks within the EU. Although norovirus is not a zoonotic agent, it has still a strong One Health element, being food and waterborne and also transmitted from person to person.

In the EU, reported norovirus outbreaks have mainly been caused by contaminated vegetables, fruits, cereals, sprouts, herbs and spices [4]. Among these products, non-heat-treated raspberries are a common source of outbreaks, as reported, e.g. in Norway [37], France [38], Sweden [39, 40], Denmark [41] and Finland [42]. One driver for this is the growing trend for eating healthy and minimally processed food, including increased consumption of, e.g. smoothies and yoghurts based on fruit or berries [43].

Raspberries receive little or no processing prior to consumption and there is thus no pathogen inactivation step before consumption [44]. This, together with liberalisation of markets, has facilitated distribution of raspberries that may be contaminated by norovirus [44]. Between 1988 and 2005, there was a 4.5-fold increase in volume of berries consumed within the EU [43]. From the late 1980s to the 2000s, there was also a 2.5-fold increase in imports of soft frozen fruits into the EU for processing [44].

Due to long survival of norovirus and low reduction in infectivity during storage, it is difficult to reduce the risk of norovirus infection from consuming berries. Freezing reduces the viability of the virus by less than one log or 90% [45], which is not enough to eliminate the risk of infection. For example, outbreak data have shown that raspberries which have been frozen for months can be the vehicle for norovirus outbreaks [40]. Processing routines, such as mixing batches from different origins during freezing and before packaging for frozen berries, may lead to large-scale cross-contamination and consequently disease outbreaks [36]. Tracing contaminated batches of raspberries following outbreaks has revealed complex distribution systems. For example, one batch of frozen raspberries may originate from more than 60 different farms, in many cases small-scale producers [41, 42]. This makes it difficult to detect the farm(s) that was the point of introduction of the agent.

It is seldom known if norovirus contamination occurred at processing, freezing, packing or during primary production, as there are knowledge gaps on the risk factors for norovirus-contaminated berries [46–48]. However, it has been suggested that viral contamination most likely takes place at the production step, more specifically during irrigation with contaminated water or during collection by food handlers’ hands, particularly if there are insufficiencies regarding hygiene conditions during picking, e.g. lack of toilets and hand-washing facilities [44, 46]. Raspberries consumed fresh are usually manually harvested due to the fragility of the plant and the berry [44]. The importance of norovirus contamination by hand contact has been confirmed in a farm-to-fork risk assessment model and a human norovirus exposure assessment [47–49].

The use of sewage-contaminated water should be avoided at all stages of the supply chain [44] and an EU regulation states that only water which does not contain micro-organisms or other harmful substances in quantities capable of affecting the health quality of food should be used during production of berries (EC Regulation 852/2004). This is challenging, as norovirus can be present in surface water [50]. Presence of norovirus can be correlated with faecal indicators such as *E. coli* [51], which emphasises the risk of contamination of irrigation water sources by sewage and effluents [44].

There is no regular monitoring of berries for the presence of norovirus in most EU member states and there are limited prevalence data on norovirus contamination of berries in the published literature. In addition, quantitative data on viral load are scarce. It is therefore not possible to provide a risk base for establishing a process hygiene criterion and food safety criterion for these foods, which has been proposed for other foods such as oysters [52]. Good agricultural practices (GAP), good hygiene practices (GHP) and good manufacturing practices (GMP) are the primary objective of operators producing berries [44].

### *Shiga* toxin-producing *Escherichia coli* in sprouts

STEC infections are the fourth most commonly reported zoonosis in the EU. While serogroup O157 continues to be the most commonly reported (42% in 2015), there appears to be an increasing trend in other serogroups and non-typable STEC strains. This is partly due to increased awareness of other serogroups and better diagnostic methods [4]. Cattle are the main recognised STEC reservoirs and bovine meat is considered to be a major source of foodborne STEC infections in humans [53]. However, in recent years some of the major outbreaks caused by STEC within the EU have been attributed to vegetables [54, 55]. Despite this, only a small fraction of fruit and vegetable samples and sprouted seed samples have been found to test positive for STEC in the EU [4].

In 2011, a unique *E. coli* strain (STEC O104:H4), which had not previously been considered to be of public health importance, was reported in Northern Germany [56]. This was the start of one of the largest STEC outbreaks ever and was followed by a similar outbreak in France in the same year [57]. The STEC O104:H4 strain differed in several ways from previously described STEC strains, e.g. with a much higher fraction of cases developing HUS and severe neurological symptoms [56]. In total, this outbreak caused more than 3800 cases of illness and more than 50 deaths [58] and involved several EU countries [59]. The causative organism was an enteroaggregative *E. coli* (EAEC) that had acquired the ability to produce Shiga toxin via horizontal gene transfer. The result was a strain with enhanced adherence factor that may have facilitated the absorption of Shiga-toxin, resulting in the severity of symptoms found in patients in this outbreak [60]. The strain had not been described in animals and only rarely in humans, suggesting a human reservoir, whereas typical STEC strains are zoonotic [61].

Initial epidemiological studies indicated fresh salad vegetables as the probable vehicle of infection [59]. In an early stage, Spanish cucumbers were declared as being the source of the outbreak [62], but this was incorrect and based on preliminary test results. This mistake resulted in economic losses amounting to over 800 million Euro for horticulture producers in several EU countries, as their products were withdrawn from the market [63]. Ultimately, organic fenugreek sprouts from seeds imported from Egypt were identified as the highly likely cause of the outbreak [62]. It is speculated that asymptomatic workers may have been the cause of seed contamination [64].

During the outbreak, appropriate risk and crisis management was delayed, as it was not possible to conduct a risk assessment. This was caused by the challenges in identifying the causative agent due to lack of methods available for the detection of STEC strain O104:H4 in the beginning of the outbreak [62]. The fact that sprouted seeds are usually an inconspicuous ingredient, and often feature as a garnish, may also have prolonged the investigation to determine the implicated source [64]. The outbreak caused economic and reputational damage not only to vegetable producers, but also to retailers and government authorities [65]. When the outbreak was over, several promotion activities were launched in order to win back consumer trust in fruit and vegetables [66].

Sprouted seeds have been identified as high-risk foods for STEC and *Salmonella* and the majority of outbreaks caused by sprouted seeds have been associated with these pathogens [67]. The largest reported outbreak associated with sprouted seeds, with over 10,000 notified cases, occurred in Japan in 1996 and was attributed to consumption of radish sprouts contaminated with STEC O157:H7 [68]. Contamination of dry seeds with bacterial pathogens is the most likely initial source of the outbreaks associated with sprouted seeds, although other routes of contamination (e.g. during production due to poor practices) may also occur [69]. The most relevant risk factors for dry seed contamination are associated with the effect of agricultural practices on seed production, storage and distribution, e.g. contaminated irrigation water and/or manure or presence of birds and rodents in storage facilities [70].

Due to the high humidity and the favourable temperature during sprouting, bacterial pathogens present on dry seeds can multiply and result in a public health risk [71]. As in production of berries, GHP and control based on hazard analysis and critical control point (HACCP) principles are crucial to avoid pathogen contamination [69, 72]. However, one concern as regards sprouting is that seeds are produced for several end-uses (e.g. edible seeds, animal feeds, oil production, horticulture) and not specifically for sprout production. Thus, the seed grower does not necessarily know whether the seed will be sold for food use as seeds or sprouts and therefore may have little incentive to follow GAPs [69]. Seeds grown for the production of sprouts for human consumption should be segregated from products intended for other uses [73]. Another concern shared with raspberry production is that seed processing, shipping and selling practices often involve mixing multiple lots of seeds of different origins, complicating traceback and providing an opportunity for cross-contamination [69]. Once present on or in seeds, pathogenic bacteria are likely to survive for extended periods of time [74]. There is so far no bactericidal step which is able to completely control contamination of seeds with bacterial foodborne pathogens acquired prior to germination [70]. However, hot water treatment is reported to be effective for disinfecting inoculated STEC O157:H7 and *Salmonella* [75].

As one of the components of a food safety management system for sprouted seeds, food safety criteria for *Listeria monocytogenes* and *Salmonella* were laid down in EU Regulation (EC) No. 2073/2005, amended together with a process hygiene criterion for *E. coli*, as a result of EFSA opinion recommending strengthened microbiological criteria [70]. An additional microbiological criterion on sprouted seeds was laid down in which absence of STEC (six serogroups, including O104) has to be proven in 25 g (n = 5) (EC 2073/2005 with amendments included 2013).

### The importance of *Listeria monocytogenes* in ready-to-eat foods

There has been an increasing trend in human listeriosis since 2008 and in 2015 it was the fifth most frequently reported zoonosis in the EU. While still relatively rare compared with campylobacteriosis and salmonellosis, human listeriosis is the most deadly zoonosis in the EU, with a hospitalisation and fatality rate of 90 and 20%, respectively, particularly among the elderly population [4].

The fact that most listeriosis cases appear to be sporadic and that the incubation period can be very long [76] makes it difficult to detect links between human cases and causative foods [77]. Since *L. monocytogenes* is ubiquitous in nature, a wide range of foodstuffs can become contaminated [77]. Most reported outbreaks within the EU have involved processed, refrigerated ready-to-eat (RTE) products of animal origin, such as delicatessen meats, smoked salmon and soft cheeses [78]. However, the list of implicated food categories associated with human listeriosis has lengthened significantly during recent years. For example, food of plant-derived origin (e.g. melon, toffee apple) or even frozen foods (e.g. ice-cream) have been implicated in outbreaks globally [79], illustrating that, under certain unexpected conditions, almost all RTE foods may have the potential to contribute to the burden of disease. In a source attribution of listeriosis in England and Wales, the major source of infection was multicomponent foods, for example sandwiches and pre-packed mixed salad vegetables [19].

Post-processing cross-contamination from equipment and the environment represents a major concern for *L. monocytogenes*, although the bacterium is inactivated by the thermal treatments used for production of some RTE foods [80, 81]. In 2006, new EU food hygiene regulations came into force recommending that food businesses manufacturing RTE foods should monitor processing areas and equipment for the presence of *L. monocytogenes* as part of their sampling schedule (EC No 2073/2005). The limit for the EU food safety criterion for *L. monocytogenes* is set at 100 CFU/g for RTE products on the market. Despite these criteria being applied, the presence of *L. monocytogenes* in RTE foods represents one of the major challenges for the food industry.

*Listeria monocytogenes* can grow at a wide pH range, at high salt concentrations and at refrigeration temperature [82]. The wide growth range and the biofilm-forming capacity allows this pathogen to subsist in the food processing plant environment, survive various food processing hurdles and proliferate in food products [83]. *Listeria monocytogenes* finds favourable growth conditions on floors, in drains and on equipment with harbourage sites (i.e. shelters due to unhygienic design) and in unhygienic or damaged materials, where strains of *L. monocytogenes* are recurrently found despite cleaning and disinfection [84]. For example, a dicing machine can sustain contamination by *L. monocytogenes* and transfer a specific PFGE type for a long time [85]. Even when using hygienically designed and well-maintained equipment and with stringent implementation of GMP, total control of *L. monocytogenes* in RTE food processing plants, including on non-food contact surfaces (e.g. drains), is extremely difficult [84]. The use of modified atmosphere packaging or anti-microbial additives, e.g. lactate [86], may prolong the shelf-life of refrigerated RTE foods, which may reduce food waste. However, it may also allow prolonged growth of *L. monocytogenes*, which is particularly important if the product is stored at abuse temperatures [87, 88]. Unsafe practices in consumers’ homes are not uncommon, e.g. it has been reported that the mean temperature in domestic refrigerators in EU countries ranges from 4 to 8 °C and the maximum temperature from 10 to 21 °C [89], with higher temperatures constituting a higher risk of *L. monocytogenes* growth.

## Antimicrobial resistance as a foodborne One Health problem

Detection of antimicrobial substances in foodstuffs is a rare event, while detection of bacteria with genes for antimicrobial resistance (AMR) is common. For example in Sweden during 2015, around one out of every 5000 samples taken from domestic food animal production tested positive for antimicrobial substances, while in import controls antimicrobials were found in one batch out of 3500 consignments of foodstuffs originating outside the EU. In contrast, monitoring of broilers at slaughterhouses in Sweden indicated that, in the period 2010–2016, between 25 and 50% of broiler carcasses were contaminated with extended spectrum beta-lactamase-producing Enterobacteriaceae (ESBL) [90]. Hence the One Health concern is foodborne consumer exposure to bacteria with genes coding for resistance to specific antibiotics.

Concerns about emerging AMR bacteria have previously been tempered by the knowledge that development of AMR in bacteria imposes a fitness cost [91]. Hence, resistant bacteria will initially have lower viability and ability to multiply than sensitive bacteria in an environment free of antimicrobials. The practical implication of the fitness cost proposition is that once the use of antimicrobials ceases, the sensitive bacteria will again prevail in competition with the resistant bacteria. As always, the picture is more complicated. Already 15 years ago, Zhang et al. [92] noted that fluoroquinolone-resistant *Campylobacter* spp. appeared to have no fitness costs compared with non-resistant *Campylobacter* spp., with the implication that ending the use of quinolones would have no impact on the presence of quinolone-resistant *Campylobacter* spp. Moreover, it appears that the use of antibiotics is correlated with higher mutation rates, and thereby higher likelihood of resistance emerging [93]. These mutations may also reduce the fitness costs associated with resistance [94].

Detection of bacteria with genes for resistance in foodstuffs is a concern because foodstuffs are efficient transmission pathways for carrying, and thereafter exposing consumers, to bacteria with genes encoding for AMR. Consequently, rapid spread of AMR bacteria can be foreseen if foodstuffs are contaminated. The AMR genes can persist either in commensals, including indicator bacteria, or on pathogens, and the genes can be exchanged between different species of bacteria along the food chain [95].

In an own-initiative opinion on AMR, the biological hazards panel of the EFSA [95] noted that resistant *Salmonella* and *Campylobacter* spp. are foodborne and causing human disease. For example, poultry meat appears to be a major source of *Campylobacter* spp. with quinolone resistance. Implicated foodstuffs for spreading cephalosporin resistance are poultry, pork and beef. Hence, food production systems must be designed to prevent the spread of resistant bacteria to consumers. Moreover, the EFSA scientists raised the concern that amongst foodborne pathogens and commensals, there is an increasing and diverse range of resistance to antimicrobial agents of human and veterinary importance. They concluded that any further spread of resistance among bacteria in foods is likely to increase human exposure and consequently the risks to public health.

In One Health discussions, AMR is usually ranked amongst the top concerns on which the veterinary and public health sides need to collaborate. The use of antimicrobials in food animals has serious negative externalities or side-effects, as it provides an excellent environment for the spread and persistence of AMR zoonotic bacteria in animal food production systems, resulting in antimicrobials used in human medicine becoming less useful. Nearly 20 years ago, Aarestrup and Wegner [96] noted that modern food animal production requires large amounts of antimicrobials and concluded there is an urgent need to implement strategies for mitigating and controlling AMR. Recently, a more pessimistic view was presented by Courvalin [97], who concluded that the development of AMR is unavoidable, but could perhaps be delayed.

## Bovine spongiform encephalopathy and One Health challenges

Bovine spongiform encephalopathy (BSE) or “mad cow disease” is a member of the group of diseases called transmissible spongiform encephalopathies (TSEs) affecting the brain and nervous system of humans and animals, all caused by abnormal forms of proteins (prions). BSE is a zoonotic disease in cattle, causing variant Creutzfeldt-Jakob disease (vCJD) in humans [98, 99]. The origin or original source of BSE is unknown, but at the end of the 1970s an established cycle of nutrients was changed in the rendering process in the UK, thereby enabling circulation and amplification of the BSE agent. The changes included feeding cattle and calves with meat-and-bone meal (MBM) of ruminant origin. The reasons for this feeding system based on recycling of nutrients included: (a) the need for high-nutrient animal feeds to increase yields in dairy production, (b) the quest for cheaper feed ingredients with high protein content, (c) reducing amounts of animal waste and by-products and thereby associated costs, and (d) food security and self-sufficiency [100]. The rendering process included sufficient heat treatment to ensure that zoonotic and animal pathogens such as *Salmonella* and classical swine fever (CSF) virus were killed. However, this treatment was not sufficient to inactivate prions.

De Koeijer [101] concluded that one infected cow could infect 15–20 other cows on average through the rendering, MBM and cow feed cycle, thereby indicating the potential of an outbreak emerging. According to the UK BSE Inquiry report [102], the first cases in cattle in the UK were noted in December 1984, while official recognition of the new disease as BSE was 2 years later. During 1987, epidemiological pathological studies were launched and these established that ruminant MBM was a risk factor for BSE, so use of ruminant MBM for feeding cattle was prohibited in 1988 [103]. At this time, BSE became notifiable, an eradication policy for cattle showing clinical symptoms was initiated and the question of whether BSE was a zoonosis was raised [104]. Studies [105, 106] later confirmed that BSE is a zoonosis. In particular, the prion protein deposited in the brain of vCJD patients was found to be indistinguishable from that of BSE affected cattle; the neuropathological changes in macaques inoculated with BSE were similar to those in vCJD patients; and transmission studies in laboratory rodents showed that the characteristics of the infectious agent in BSE and vCJD were remarkably similar [105, 106]. In 1989, specified bovine offal (SBO, e.g. brain, spinal cord and eyes, but later extended to distal ileum and spleen) was prohibited for human consumption, and petfood manufacturers voluntarily ceased the use of SBO [104].

Other measures that were implemented in the 1980s included culling of cows showing clinical symptoms and destruction of the carcasses, and the removal of specified risk material (SRM, formerly SBO) in all cattle. Ducrot et al. [107] noted that the number of BSE-infected cows decreased for each age cohort born 1988 and thereafter, indicating the efficiency of the control measures. On the other hand, no measure appeared sufficient to eliminate the apparent increase in BSE incidence amongst cows as measured by clinical symptoms.

The peak of the epidemic in the UK was observed in 1992/93, which was around 4 years after the first control measures were implemented. It thus appeared that BSE had an incubation period of four to 6 years [108]. This delay in clinical onset of symptoms resulted in other EU countries not implementing control measures and making the mistake of viewing BSE as a UK-only problem during 1988–1993. Yet during this period, the rest of Europe imported ruminant MBM from the UK as a possible ingredient for poultry and pig feed production [102, 109]. Moreover, calves and heifers, a number of which were most likely infected with the BSE agent, were exported from the UK. This was the driver for the next wave of BSE in several EU countries [107]. BSE cases were subsequently found in Ireland in 1989, in Switzerland in 1990, in France in 1991 and in Denmark in 1992 [110, 111]. National bans on feeding bovine MBM to cattle became implemented more widely in Europe. However, it was only in 1994 that the EU imposed a general ban on feeding mammalian MBM to cattle. This was extended in 2001 to a ban on feeding any MBM to any food animals in the EU [107]. A BSE geographical risk assessment predicted that countries which had imported cattle MBM from the UK were at high risk of incubating BSE, in particular if their rendering and feeding practices enabled the BSE agent (prions) to circulate [112, 113]. Thus, for animal diseases with long incubation periods, the assumption of disease freedom in a region or country is based not only on the absence of clinical disease, but also on the absence of exposures or risk factors in periods commensurate with the incubation period of that particular disease [114].

From 1995 to 1997, 21 human cases of vCJD were reported [115], nearly all in the UK. From October 1996 to March 2011, 175 cases of vCJD were reported in the UK and 49 cases in other countries [116]. A noteworthy feature was the young age at the onset of symptoms, as the youngest case was 16 years and median age was 29 years. Two modelling studies predicted that 200,000 and 1 million people, respectively, in the UK were incubating vCJD [117, 118]. These modelling results were substantiated to some extent 15 years later by Gill et al. [119], who studied the prevalence of abnormal prion protein in the human appendix and found a prevalence of one carrier per 2000 people, or around 30,000 carriers in total in the UK.

The control and risk management of BSE took place in an environment with political disputes—the BSE crisis of 1996 [120]. The priority of the EU was to protect consumer confidence in the official control and safety of food produced and sold in the EU. In brief, further control measures that were seen as draconic, but in retrospect necessary, were needed to stop the epidemic by breaking the cycle of pathogens in the cattle food and feed chain and thereby also protecting the consumer. These measures included a total ban on animal MBM in feed intended for food animals all over EU, testing at slaughter of all cattle older than 30 months for prions and, in the UK, the destruction of all cattle above 30 months of age and testing of all fallen stock. In some cases whole cattle herds were slaughtered and the carcasses destroyed when one BSE case was found, in particular in the early days of the epidemic. The most stringent measures applied to countries where the geographical risk assessment indicated a high risk of BSE. The control measures were efficient, but not sufficient to control and eliminate the public health risk and consumer concerns [120]. One successful action on the EU level was the development of two roadmaps for control of prion diseases, including BSE [121, 122].

## Conclusions

There are several important lessons to be learnt from the cases presented above to illustrate One Health and food safety challenges in Europe. The cases, caused by different combinations of pathogens/food commodities, are examples of the importance of having sufficient knowledge of the incidence and burden of foodborne diseases within Europe. This is particularly true for the non-zoonotic foodborne diseases that are not included in any EU reporting. Food safety resources need to be allocated where they result in the largest One Health benefits and risk reductions and these can be prioritised using a combination of different measures on health risks. The One Health challenges include developing similar measurable metrics for animal health and welfare and environmental health that enable comparisons. Currently, this has to be solved on a case-by case basis.

It can also be discussed whether a risk-based or a hazard-based approach to control foodborne infections should be used. From a public health perspective, it might be tempting to focus on specific hazards, but the greatest health benefits will be achieved if a risk-based approach is used. One example of a hazard-based approach is the focus on controlling *Salmonella* in foodstuffs. This has generally been successful, but it has not necessarily improved the food safety situation, as *Campylobacter* spp. infections have been the most common foodborne zoonosis in recent years. The examples presented in this paper show that control in primary production and processing is crucial for reducing the occurrence of pathogens in the food web. The current increase in consumption of raw or minimally processed food commodities poses extra challenges for products requiring a high level of manual handling.

From the case describing AMR, it can be concluded that the best strategy is to delay and if possible prevent the emergence and subsequent dissemination of resistant bacteria or resistance genes. Consequently, liberal use of antimicrobial substances in veterinary medicine to treat food and companion animals is not sustainable in the long run. Thus antimicrobial drugs must not be used to compensate for substandard rearing facilities and animal welfare. Instead, preventative medicine must be improved, including better biosecurity and reinforcement of animal health and welfare within production systems, and there must be better access to vaccines to protect against infections and more animal breeding programmes aimed at robustness and resilience.

The BSE case illustrates the importance of including a One Health perspective at an early stage in an outbreak or when controlling foodborne diseases, and also when designing circular food systems. For example, the ability to spread and the magnitude and severity of the BSE epidemic was only fully grasped when the One Health approach was applied. The One Health approach revealed that important aspects of the BSE epidemic, such as food safety, public health and the ability of the BSE agent to spread through the food and feed chains, were overlooked. Hence in retrospect and regret, one could conclude that timely preventive and prophylactic measures were not implemented, thus enabling the spread of BSE all over Europe and greater exposure of consumers.

All this is a part of the larger challenge of feeding 11 billion people with safe and wholesome food without enlarging the environmental footprint of food production and consumption. We foresee that this challenge will test food security and safety systems to their limits and sometimes beyond. The key will be to find working solutions that consider several competing aims, in other words the overall best solutions. Moreover, food safety, nutrition and security are complementary and not competing aims, and must be pursued simultaneously.

Future achievements in food safety, public health and welfare within Europe will largely depend on how well politicians, researchers, industry, national agencies and other stakeholders manage to collaborate. This review shows that there is a high degree of complexity in the food web. Without close One Health collaboration between all parties, it will be difficult to solve the challenges of tomorrow and find the best solutions.

## Abbreviations

AMR: antimicrobial resistance
BSE: bovine spongiform encephalitis
DALY: disability adjusted life years
EFSA: European Food Safety Authority
EU: European Union
GAP: good agricultural practices
GHP: good hygiene practices
GMP: good manufacturing practices
HACCP: hazard analysis and critical control point
HUS: haemolytic uraemic syndrome
MBM: meat-and-bone meal
QUALY: quality adjusted life years
RTE: ready-to-eat
SBO: specified bovine offal
STEC: shiga toxin-producing *Escherichia coli*
vCJD: Creutzfeldt-Jakob disease
WHO: World Health Organisation
